# MicroRNA-130a attenuates cardiac fibrosis after myocardial infarction through TGF-β/Smad signaling by directly targeting TGF-β receptor 1

**DOI:** 10.1080/21655979.2022.2033380

**Published:** 2022-02-21

**Authors:** Yu Feng, Yintu Bao, Jiaxing Ding, Huili Li, Wei Liu, Xuehua Wang, Hongquan Guan, Zhijian Chen

**Affiliations:** Department of Cardiology, Union Hospital, Tongji Medical College, Huazhong University of Science and Technology, Wuhan, China

**Keywords:** Cardiac fibrosis, myocardial infarction, miR-130a, cardiac fibroblast, TGFBR1

## Abstract

Cardiac fibrosis is a common pathophysiological change associated with myocardial infarction (MI), and while there is evidence that miR-130a plays an important role in a variety of fibrotic diseases, its role in the cardiac fibrosis during MI is unclear. Our study aimed to assess miR-130a’s ability to modulate cardiac fibrosis post-MI and uncover its potential molecular mechanisms. miR-130a was significantly downregulated in infarcted myocardium and hypoxic cardiac fibroblasts (CFs), whereas TGF-β, α-SMA, collagen 1 (Col-1), and TGF-β receptor 1 (TGFBR1) were upregulated. We transfected mice with AAV-9 carrying miR-130a and found that miR-130a overexpression statistically improved cardiac function and reduced the area of cardiac fibrosis in mice post-MI. Eukaryotic transcriptome sequencing and dual-luciferase reporter assay results verified that *Tgfbr1* was a target gene of miR-130a. miR-130a inhibition heightened Col-1, α-SMA, and TGFBR1 expressions and Smad3 phosphorylation levels in CFs; however, these increments were suppressed by the overexpression of miR-130a. Meanwhile, co-transfection with TGFBR1 weakened miR-130a’s ability to inhibit α-SMA and Col-1 expression. These findings suggest that miR-130a exerts antifibrotic properties by directly targeting TGFBR1 to regulate TGF-β/Smad signaling and inhibit the conversion of CFs to myofibroblasts. Thus, miR-130a is a promising therapeutic target for alleviating cardiac fibrosis.

## Graphical Abstract

**Figure:**
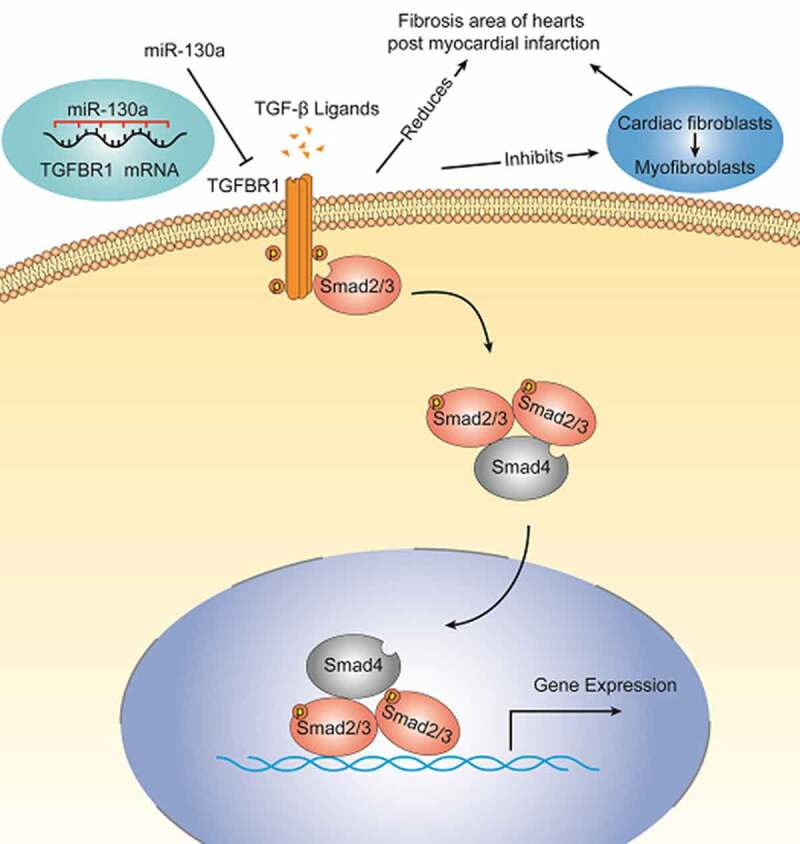

## 1 Introduction

Cardiovascular diseases are currently the leading causes of mortality and morbidity among non-communicable diseases worldwide [1,[2], with acute myocardial infarction one of the most severe cardiovascular events and myocardial infarction (MI) the most common cause of heart failure [3]. Severe myocardial interstitial fibrosis post-MI leads to left ventricular dysfunction, resulting in heart failure development [4].

Cardiac fibroblasts (CFs) account for approximately 11% of adult mice heart cells and play a crucial role in cardiac fibrosis, an essential component of cardiac remodeling [5,6]. TGF-β/Smad signaling has a key function in the regulation of CFs and the development of fibrosis [7,8]. TGF-β is markedly upregulated in experimental models of myocardial infarction. Members of the TGF-β superfamily transduce signals from the membrane to the nucleus through different combinations of transmembrane type I and type II serine/threonine receptors and their downstream effectors, Smad proteins. Active TGF-β binds to and phosphorylates TGF-β receptor 2 (TGFBR2) and TGF-β receptor 1 (TGFBR1), with the phosphorylation of TGFBR1 activating the type I receptor kinase domain, which then propagates intracellular signals downstream through Smad proteins, vital components of TGF-β signaling. TGF-β stimulation induces the conversion of fibroblasts into myofibroblasts and enhances the synthesis of extracellular matrix proteins, promoting post-infarction ventricular remodeling [9].

MicroRNAs (miRNAs) are small, non-coding RNAs that post-transcriptionally regulate the expression of target genes by binding to their 3’ UTRs [10]. They participate in multiple pathophysiological processes in cardiovascular diseases, including arrhythmias, heart failure, myocardial hypertrophy, and atherosclerosis [11–15]. In chromosomes 11 and 22 of the miR-130 family, paralogous miRNA sequences, miR-130a and miR-130b are situated, respectively [16]. The target genes miR-130a act on are mainly related to protein phosphorylation, cell activation, and gene transcription [17–19]. Recently, increasing evidence has demonstrated that aberrantly expressed miR-130a is considered to be a regulator in some fibrosis diseases. It has been showed that miR-130a plays an important role in lung fibrosis [20], renal fibrosis [21], and liver fibrosis [22].

Currently, it has been reported that mir-130a could target PDE4D to alleviate apoptosis of cardiomyocytes and improve cardiac function post-MI [23]. However, miR-130a’s involvement in cardiac fibrosis post-MI has not been elucidated. Therefore, in this study, we will investigate the modulatory effect of miR-130a on cardiac fibrosis post-MI by constructing miR-130a AAV-9 vectors delivered to mouse hearts. We hypothesize that miR-130a might play a role in cardiac fibrosis, and hope that this research will provide a new potential target for the treatment of cardiac fibrosis.

## 2 Materials and methods

### 2.1 Experimental animals

C57BL/6 J male mice, aged 8–10 weeks, were purchased from Charles River (Beijing, China) and housed in specific pathogen-free conditions. All animal studies were performed in accordance with the National Institutes of Health guidelines (NIH Publications No. 8023, revised 1978) and approved by the Animal Care and Utilization Committee of Huazhong University of Science and Technology.

### 2.2 Induction of myocardial infarction

MI was instigated by ligating the left anterior descending artery, as described previously [24]. Briefly, mice were anesthetized with 5.0% isoflurane, intubated, and ventilated with room air using a rodent ventilator (SHANGHAI ALCOTT BIOTECH CO, ALC-V8S). Anesthesia was maintained by providing 1.5–2% isoflurane, driven by 100% oxygen flow, to rats via inhalation. Body temperature was kept at 37°C using a heating pad. With conditions primed for the procedure, the skin was incised, and the hearts were exposed through the left thoracotomy in the fourth intercostal space. The left anterior descending (LAD) coronary artery was then permanently ligated with a 6–0 silk ligature, after which the skin was closed up, anesthesia was discontinued, and the animals were allowed to recover in pre-warmed cages. The survival rate of the mice after surgery is about 90%.

### 2.3 Western blot

Total protein samples were collected from heart tissues or CFs. Lysate preparation: (RIPA: PMSF: protease inhibitor: phosphatase inhibitor = 100:1:2:2). The following reagents were used: RIPA (P0013C, Beyotime), PMSF (ST506, Beyotime), Protease and phosphatase inhibitor (P1045, Beyotime). The following primary antibodies were used: anti-α-SMA (Proteintech, 14,395-1-AP), anti-TGF-β1 (Proteintech,21,898-1-AP), anti-TGFBR1 (Abcam, ab235178), anti-collagen 1 (Col-1) (Proteintech,14,695-1-AP), anti-p-Smad3 (Cell Signaling Technology, #9520), anti-Smad3 (Cell Signaling Technology, #9523), and anti-GAPDH (Proteintech,60,004-1-lg). Goat anti-rabbit antibody (Ant Gene, ANT020) and Goat anti-mouse antibody (Ant Gene, ANT019) were applied as secondary antibodies.The following primary antibodies were used: anti-α-SMA (Proteintech, 14,395-1-AP), anti-TGF-β1 (Proteintech,21,898-1-AP), anti-TGFBR1 (Abcam, ab235178), anti-Col-1 (Proteintech,14,695-1-AP), anti-p-Smad3 (Cell Signaling Technology, #9520), anti-Smad3 (Cell Signaling Technology, #9523), and anti-GAPDH (Proteintech,60,004-1-lg). Goat anti-rabbit antibody (Ant Gene, ANT020) and Goat anti-mouse antibody (Ant Gene, ANT019) were applied as secondary antibodies.

### 2.4 RNA isolation and rt-PCR

RNA was extracted from heart tissues and cells using Trizol (Takara). The isolated RNA was then reverse transcribed into cDNA using reverse transcriptase with the cDNA Synthesis Kit (RR036A, Takara). miRNA was reverse transcriptase with the cDNA Synthesis Kit (RR037A, Takara). cDNA was amplified with SYBR Premix Ex Taq Kit (RR420A, Takara) on the CFX96 Real-Time PCR detection system (Bio-Rad) according to manufacturer’s protocol. The relative abundance of miRNA was normalized to the small nuclear RNA U6, and the expression of the genes were normalized to GAPDH. The relative amounts of the miRNAs and genes were measured using the 2-ΔΔCt method. The PCR primers were synthesized by Shanghai Shenggong Bioengineering Co. (Shanghai, China). The primers used are listed in Table 1.

**Table 1.** Primer sequences

Genes	Forward 5′→3′	Reverse 5′→3′
**miR-130a**	CGCGCAGTGCAATGTTAAAA	AGTGCAGGGTCCGAGGTATT
**Col-1**	GCCTAGCAACATGCCAATATTT	GAATACTGAGCAGCAAAGTTCC
**TGFBR1**	ATATCTGCCATAACCGCACTG	CTGAAATGAAAGGGCGATCTAGT
**TGF-β1**	TCAGACATTCGGGAAGCAGT	TGACGTCAAAAGACAGCCAC
**α-SMA** **GAPDH** **U6**	GCGTGGCTATTCCTTCGTGACTAC TGACAAGCTTCCCATTCTCG CTCGCTTCGGCAGCACA	CGTCAGGCAGTTCGTAGCTCTTC GTGAAGGTCGGTGTGAACG AACGCTTCACGAATTTGCGT

**miR-130a-stem-loop**: GAGGTATTCGCACTGGATACGACATGCCC.

### 2.5 Assessment of cardiac function and the area of fibrosis

A Vevo 2100 high-resolution microimaging system (VisualSonics, Toronto, Canada) with a 30 MHz transducer was used. The area of fibrosis was evaluated using Masson’s trichrome method [25] and calculated as the ratio of fibrotic tissue to total left ventricular area, as described previously [26].

### 2.6 Transfection of mice with adeno-associated virus 9 (AAV9)

A cardiac-specific miR-130a overexpression model was created: an AAV9-U6-mmu-mir-130a virus (an AAV9 with a U6 promoter) was injected into the tail vein of mice 2 weeks before MI induction (100 μL for each mouse in the MI+H-miR-130a group). Mice in the MI+NC group were inoculated with negative control AAV9 (NC-AAV9). The AAV9 used was synthesized by Hanbio Biotechnology Co (Shanghai, China). Specific information is available in supplementary 1.

### 2.7 Culture and transfection of CFs

Primary neonatal mouse CFs were isolated and cultured, as described previously [27]. Adenoviral vectors encoding the mouse miR-130a gene (miR-130a-Adv), negative control (NC-Adv), miR-130a sponge, and TGFBR1-expressing vectors were constructed, packaged, and purified at Hanbio Biotechnology Co. (Shanghai, China). For treatment in hypoxic conditions, CFs were starved in a serum-free medium for 24 h and placed in a hypoxic chamber at a constant temperature of 37°C in 93% N2‚ 5% CO_2_, and 2% O_2_ for 24 h.

### 2.8 Luciferase assay

Using restriction enzyme site primers, a mouse wild-type TGFBR1 3’ UTR fragment containing the conserved binding site of miR-130a was generated utilizing PCR and cloned into the pMIR vector referred to as wild-type TGFBR1 3’ UTR. The mutant TGFBR1 3’ UTR was synthesized by mutating the miR-130a binding site of TGFBR1 3’ UTR wild-type (wt) and inserted into the equivalent reporter vector. Human 293 T cells were co-transfected with 3’ UTR luciferase reporter vectors containing TGFBR1 [wt or mutant (mu)] and miR-130a fragment or miR-NC. Luciferase activity was detected using the dual-luciferase reporter assay detection system (Hanbio Biotechnology Co).

### 2.9 Eukaryotic transcriptome (with reference genome) sequencing analysis

Eukaryotic transcriptome (with reference genome) sequencing analyses were carried out by Shanghai Majorbio Bio-pharm Technology Co.,Ltd. Total RNA was extracted from the tissue using Trizol Reagent (Plant RNA Purification Reagent for plant tissue) according the manufacturer’s instructions (Invitrogen) and genomic DNA was removed using DNase I (TaKara). RNA-seq transcriptome librariy was prepared following TruSeqTM RNA sample preparation Kit from Illumina (San Diego, CA) using 1 μg of total RNA. The raw paired end reads were trimmed and quality controlled by SeqPrep and Sickle with default parameters. Then clean reads were separately aligned to reference genome with orientation mode using HISAT2 software. The mapped reads of each sample were assembled by StringTie in a reference-based approach. To identify DEGs (differential expression genes) between two different samples, the expression of each transcript was calculated according to the transcripts per million reads (TPM) method. RSEM was used to quantify gene abundances. Essentially, differential expression analysis was performed using the DESeq2/DEGseq/EdgeR with Q value ≤ 0.05, DEGs with |log2FC|>1 and Q value ≤ 0.05 (DESeq2 or EdgeR) /Q value ≤ 0.001 (DEGseq) were considered to be significantly different expressed genes). In addition, functional-enrichment analysis including GO and KEGG were performed to identify which DEGs were significantly enriched in GO terms and metabolic pathways at Bonferroni-corrected P-value ≤0.05 compared with the whole-transcriptome background. GO functional enrichment and KEGG pathway analysis were carried out by Goatools and KOBAS. All the alternative splice events that occurred in our sample were identified by using recently releases program Rmats. Only the isoforms that were similar to the reference or comprised novel splice junctions were considered, and the splicing differences were detected as exon inclusion, exclusion, alternative 5′, 3′, and intron retention events.

### 2.10 Statistical analysis

Data were presented as mean ± SD. The statistical significance between the two experimental groups was assessed by a two-tailed Student t-test. When comparing more than 2 different groups, one-way ANOVA followed by Sidak’s post hoc test and Dunnett post hoc test was used. Graph drawing and statistical analysis were performed with GraphPad Prism v.7.0. p < 0.05 was considered statistically significant.

## 3 Results

In this study, male mice were selected and randomly divided into four groups: sham group, MI group, MI+NC group, and MI+miR-130a group. We explored the effect of miR-130a on cardiac fibrosis by constructing miR-130a AAV-9 vectors delivered to mouse hearts. Meanwhile, primary neonatal mouse CFs were transfected with adenovirus carrying miR-130a to investigate the specific molecular mechanism of the antifibrotic effect of miR-130a. The expression of miR-130a, TGF-β, TGFBR1, p-Smad3, t-Smad3, α-SMA and collagen 1 (Col-1) in heart tissues and CFs were tested. We found a crucial role of miR-130a in the development of cardiac fibrosis post-MI.

### 3.1 miR-130a was down-regulated in infarcted myocardium and hypoxic CFs

**Figure 1.**
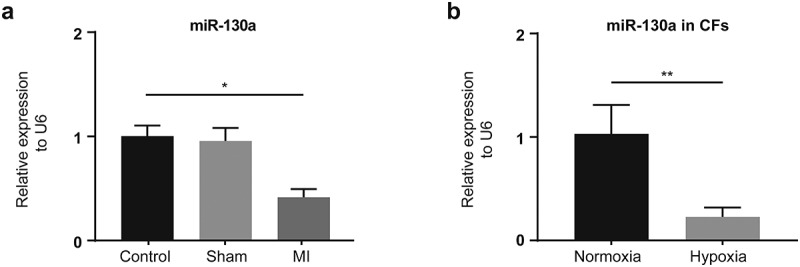
**miR-130a expression in infarcted myocardium and hypoxic CFs**. (a) miR-130a levels in the control, sham, and MI groups, as determined with rt-PCR. (n = 6 per group). (b) miR-130a levels in CFs under normoxia and hypoxia, as determined with rt-PCR. (n = 6 per group).

Our examination of the expression of miR-130a in the control, sham, and MI groups and infarcted myocardium in the hearts of mice obtained from the MI group 14 days post-MI yielded diminished miR-130a levels in infarcted myocardium (Figure 1a). Besides, the expression of miR-130a was reduced in hypoxia-treated CFs (Figure 1b).

*P < 0.05; **P < 0.01. Data are expressed as mean ± SD. *P < 0.05, unpaired t-test was used in **b**; one-way ANOVA was used in **a**. MI: myocardial infarction

### 3.2 Cardiac fibrosis was activated in mice post-MI

**Figure 2.**
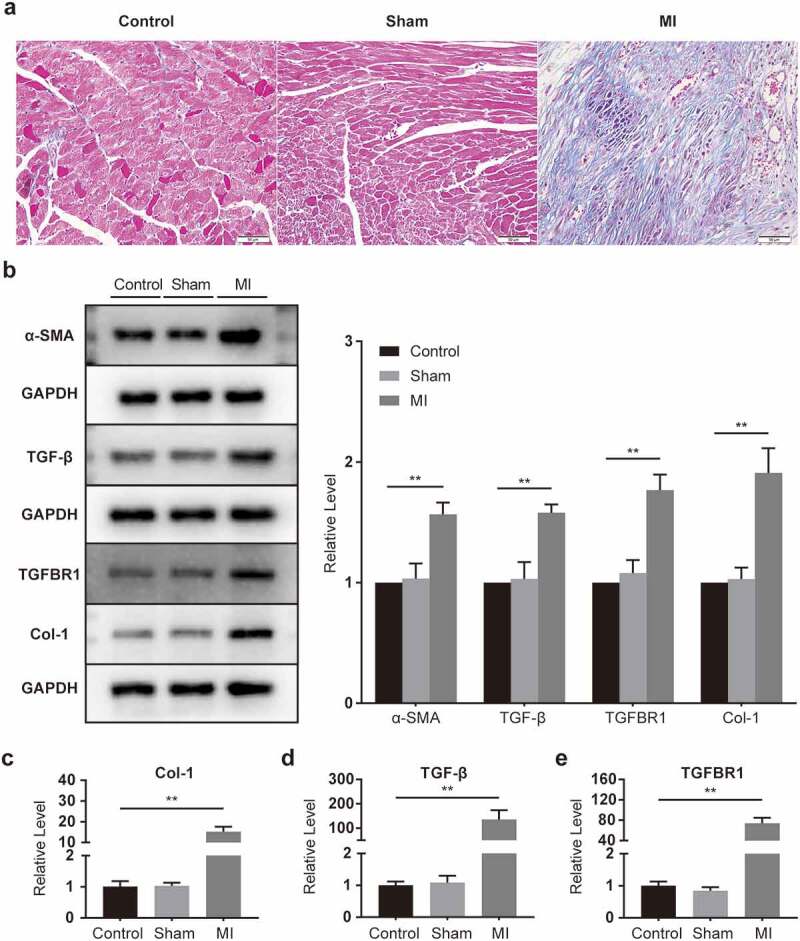
**Fibrosis activation in infarcted myocardium**. (a) Representative images of mouse heart sections (10 × 40 magnification of 400×) (n = 5 per group, Bar = 50 μm). (b) α-SMA, TGF-β, TGFBR1, and Col-1 protein levels, as determined with Western blot (n = 6 per group). (c-e) Col-1, TGF-β, and TGFBR1 mRNA levels, as determined with rt-PCR (n = 6 per group).

In the assessment of pathological changes in the heart with the help of Masson trichromatic staining, collagen deposition in the infarcted area increased substantially compared to those in the control and sham groups (Figure 2a). Western blot results showed that α-SMA, TGF-β, TGFBR1, and Col-1 were conspicuously elevated in infarcted myocardium (Figure 2b), as were the mRNA expressions of Col-1, TGF-β, and TGFBR1 (Figure 2c-e). The results showed that the hearts of mice underwent significant fibrosis after the surgery.

*P < 0.05; **P < 0.01. Data are expressed as mean ± SD. *P < 0.05, one-way ANOVA was used. MI: myocardial infarction

### 3.3 miR-130a alleviated the impairment of cardiac function and condensed the area of cardiac fibrosis in mice post-MI

**Figure 3.**
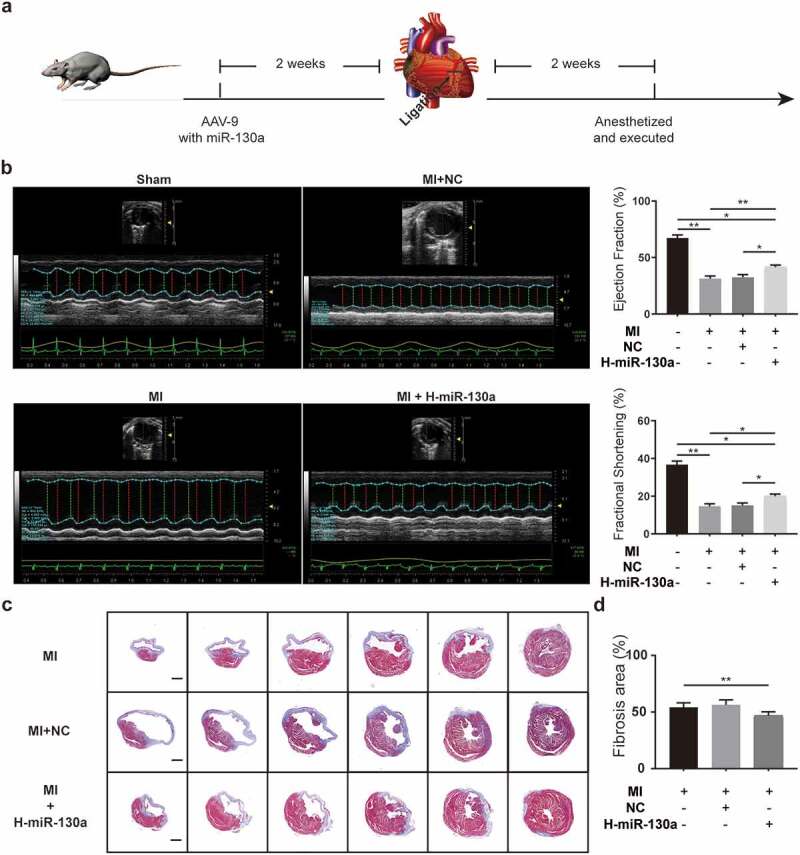
**AAV-9 transfection and measurement of cardiac function and cardiac fibrosis area**. (a) Experimental procedure and timeline of surgery and transfection of AAV-9. (b) Cardiac function quantification on day 14 post-MI, as determined with echocardiography (n = 6 per group). (c-d) Measurement of fibrosis area 2 weeks post-MI, as determined with Masson’s trichrome method (n = 6 per group, bar = 500 μm).

To determine the impact of miR-130a on cardiac fibrosis post-MI, we injected saline, negative control AAV9, and miR-130a-AAV9 into various groups of mice through the tail vein. Two weeks after the injection, MI operations were done on the mice. Cardiac function was assessed on day 14 post-MI using echocardiography (Figure 3a). According to the results (Figure 3b), cardiac function was markedly impaired in mice post-MI compared to the sham group, as evidenced by a notable decrease in ejection fraction (EF) and fractional shortening (FS). Mice with high cardiac miR-130a expression post-MI exhibited improved EF and FS relative to mice in the MI group and MI+NC (MI with negative control AAV9) group. Measuring the sizes of the areas of fibrosis in different groups of mice revealed that mice expressing high amounts of miR-130a had smaller fibrosis areas (Figure 3c,d), which were consistent with better cardiac function.

*P < 0.05; **P < 0.01. Data are expressed as mean ± SD. *P < 0.05, one-way ANOVA was used. MI: myocardial infarction; MI+NC: myocardial infarction + negative control AAV9; MI+H-miR-130a: myocardial infarction + high miR-130a expression.

### 3.4 miR-130a directly acted on the 3’ UTRs of TGFBR1 mRNA

**Figure 4.**
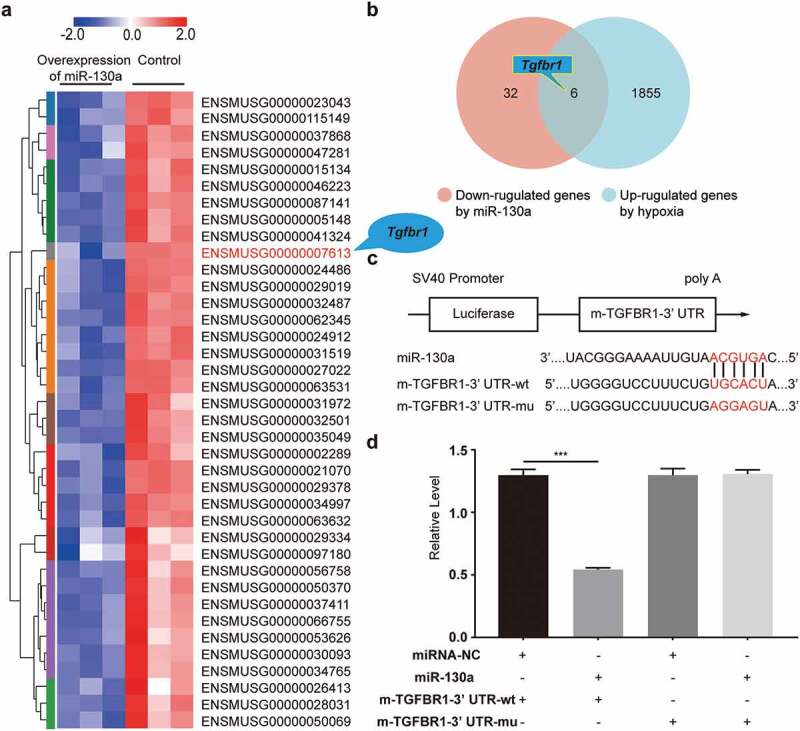
**Validation of *Tgfbr1* as a target gene of miR-130a**. (a) A heat map of the 38 genes from the miR-130a overexpression and control groups. (b) A Venn diagram showing the overlap between potential miR-130a target genes and hypoxia-regulated genes. (c) Bioinformatics analyses of the predicted interactions of miR-130a with its binding sites on TGFBR1 mRNA 3’ UTR. (d) Luciferase activity, as determined with the dual-luciferase reporter assay.

To identify the target genes through which miR-130a exerted its effects on CFs, we used eukaryotic transcriptome (with reference genome) sequencing analysis to predict miR-130a-3p target genes associated with cardiac fibrosis. We found that 6 genes (*Sco2, Acta1, Htr2a, Tgfbr1, Trib1, Asb5*) were repressed by miR-130a and increased in hypoxic CFs (Figure 4b). Moreover, per computational predictions, the seed sites of miR-130a matched with TGFBR1 3’ UTR (Figure 4c).

*P < 0.05; **P < 0.01; ***P < 0.001. Data are expressed as mean ± SD. *P < 0.05, one-way ANOVA was used. miR-130a: plasmid with miR-130a; miRNA-NC: plasmid with miR-130a negative control; m-TGFBR1-3’ UTR-wt: plasmid with wild-type mouse TGFBR1 3’ UTR; m-TGFBR1-3’ UTR-mu: plasmid with mutant mouse TGFBR1 3’ UTR.

To ensure that miR-130a directly binds to the 3’ UTRs of TGFBR1 and induces translational inhibition, we conducted the dual-luciferase reporter assay, which we divided into two parts (Figure 4d). First, the luciferase vector carrying the 3’ UTR-wt of TGFBR1 was co-transfected with miR-130a (miR-130a + m-TGFBR1-3’ UTR-wt), yielding strikingly diminished luciferase activity compared to the control (miRNA-NC + m-TGFBR1-3’ UTR-wt), which indicated that miR-130a combined with TGFBR1 3’ UTR. Additionally, there was no difference in luciferase activity between the plasmid with miR-130a negative control + the plasmid with TGFBR1 3’ UTR-mu (miRNA-NC + m-TGFBR1-3’ UTR-mu) and the plasmid with miR-130a + the plasmid with TGFBR1 3’ UTR-mu (miR-130a + m-TGFBR1-3’ UTR-mu), implying that miR-130a did not combine with the mutant TGFBR1 3’ UTR. These results confirm that *Tgfbr1* is one of the target genes of miR-130a.

### 3.5 The impact of miR-130a on TGF-β/Smad signaling in CFs under normoxia

**Figure 5.**
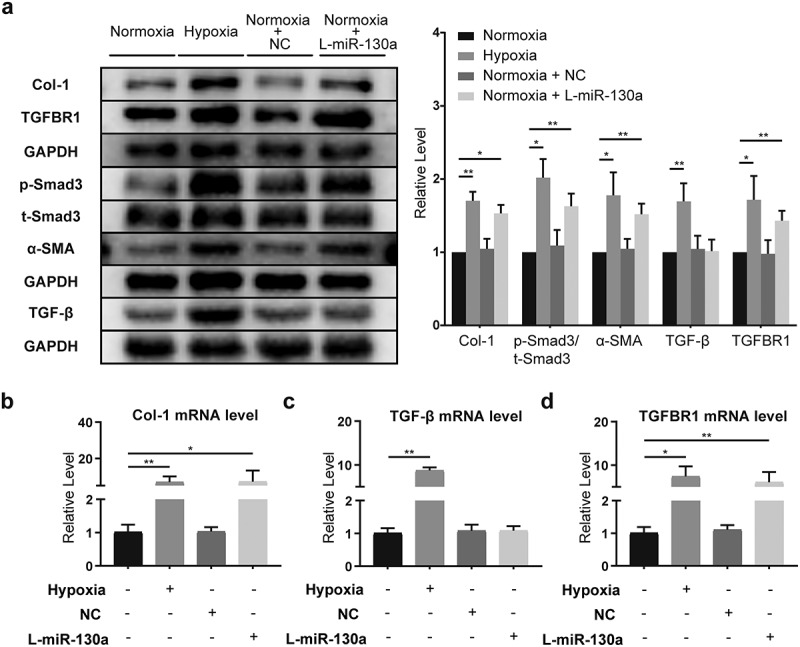
**miR-130a’s impact on TGF-β/Smad signaling in CFs under normoxia**. (a) Col-1, TGFBR1, p-Smad3, t-Smad3, α-SMA, and TGF-β protein levels in CFs under normoxia, as determined with Western blot (n = 6 per group). (b-d) Col-1, TGF-β, and TGFBR1 mRNA levels in CFs under normoxia, as determined with rt-PCR (n = 6 per group).

We examined hypoxia’s influence on cardiac fibroblasts and found that hypoxia triggered the activation of TGF-β/Smad signaling, with considerably enhanced TGF-β, TGFBR1, α-SMA, and Col-1 protein expressions and a significant increase in p-Smad3/t-Smad3 (Figure 5a). Meanwhile, rt-PCR results showed that the mRNAs of Col-1, TGF-β, and TGFBR1 in CFs were amplified under hypoxia (Figure 5b-d).

*P < 0.05, **P < 0.01. Data are expressed as mean ± SD. *P < 0.05, one-way ANOVA was used. NC: negative control adenovirus; L-miR-130a: low miR-130a expression; H-miR-130a: high miR-130a expression.

In order to determine the specific effects of miR-130a on CFs, we transfected primary neonatal mouse CFs with negative control adenovirus (NC) and used miR-130a-Adv and miR-130a sponge to upregulate and downregulate miR-130a expression, respectively. Notably, to eliminate the influences of potential confounding factors of hypoxia, we scrutinized the activation of TGF-β/Smad after transfection with miR-130a sponge under normoxic conditions. CFs transfected with miR-130a sponge harbored elevated Col-1, α-SMA, and TGFBR1 expressions and heightened p-Smad3/t-Smad3 (Figure 5a). Rt-PCR analyses also revealed augmented Col-1 and TGFBR1 levels (Figure 5b,d); however, TGF-β protein and mRNA expressions were not statistically altered (Figure 5a,c).

### 3.6 The impact of miR-130a on TGF-β/Smad signaling in CFs under hypoxia

**Figure 6.**
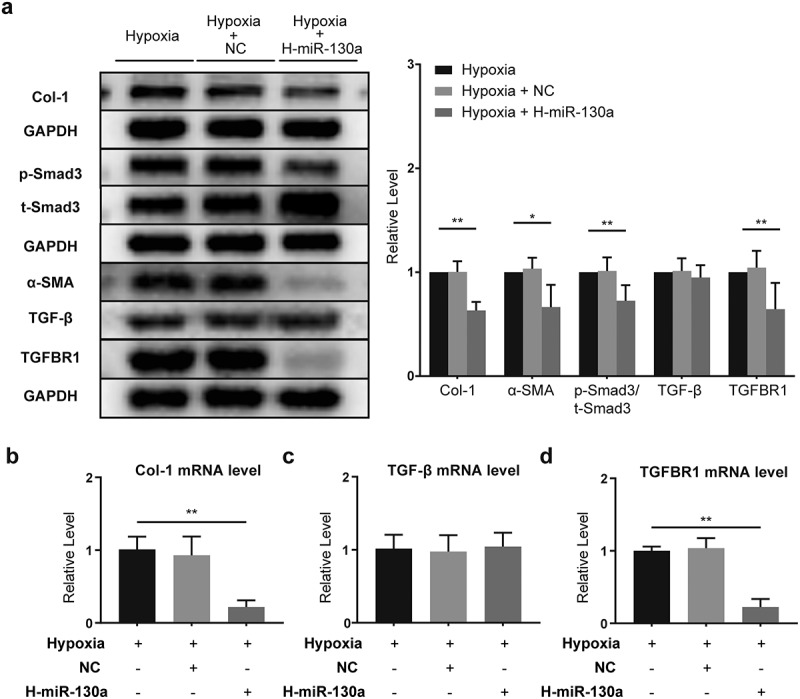
**miR-130a’s impact on TGF-β/Smad signaling in CFs under hypoxia**. (a) Col-1, p-Smad3, t-Smad3, α-SMA, TGF-β, and TGFBR1 protein levels in CFs under hypoxia, as determined with Western blot (n = 6 per group). (b-d) Col-1, TGF-β, and TGFBR1 mRNA levels in CFs under hypoxia, as determined with rt-PCR (n = 6 per group).

To investigate the effects of miR-130a on the TGF-β/Smad signaling under hypoxic conditions, we infected CFs with adenovirus carrying miR-130a in hypoxia. Under hypoxia, TGF-β/Smad signaling was inhibited in CFs transfected with miR-130a-Adv, as demonstrated by the down-regulation of Col-1, α-SMA, and TGFBR1 protein expressions and decreased p-Smad3/t-Smad3 (Figure 6a). TGF-β levels were not appreciably altered (Figure 6a). Col-1 and TGFBR1 mRNA levels shrunk (Figure 6b,d), but TGF-β mRNA expression remained unchanged (Figure 6c).

*P < 0.05, **P < 0.01. Data are expressed as mean ± SD. *P < 0.05, one-way ANOVA was used. NC: negative control adenovirus; L-miR-130a: low miR-130a expression; H-miR-130a: high miR-130a expression.

### 3.7 TGFBR1 attenuated miR-130a-impaired activation and collagen deposition in CFs

**Figure 7.**
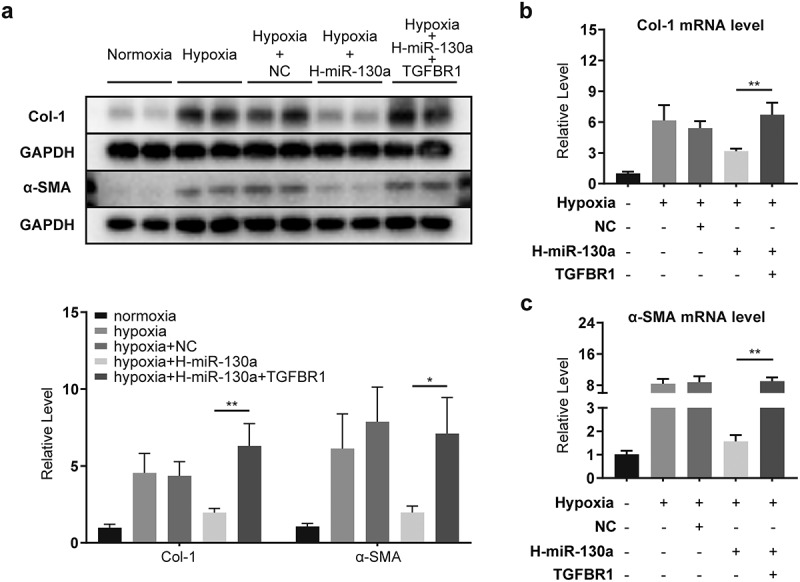
**Attenuation of the influence of miR-130a by co-transfection with TGFBR1**. (a) Col-1 and α-SMA protein levels, as determined with Western blot. (b-c) Col-1 and α-SMA mRNA levels, as determined with rt-PCR.

To assess whether TGFBR1 was a key functional target of miR-130a in CFs, we co-transfected the CFs with miR-130a and TGFBR1-expressing vectors. Comparable to the results above in which a significant reduction in miR-130a culminated in markedly elevated α-SMA and Col-1 in infarcted myocardium (Figure 2b), miR-130a inhibition in CFs resulted in amplified α-SMA and Col-1 (Figure 5a); however, these were suppressed by miR-130a overexpression (Figure 6a). Our co-transfection of CFs with miR-130a mimics and TGFBR1-expressing vectors yielded considerably higher α-SMA (Figure 7a,c) and Col-1 (Figure 7a,b) protein and mRNA expressions than miR-130a transfection alone. These results suggest that miR-130a subdued the conversion of CFs to myofibroblasts and collagen generation via targeting TGFBR1.

*P < 0.05; **P < 0.01. Data are expressed as mean ± SD. *P < 0.05, one-way ANOVA was used. NC: negative control; H-miR-130a: high miR-130a expression

## 4 Discussion

Fibrotic scar tissues replace damaged myocardium after MI due to the minimal regenerative capacity of cardiomyocytes in the adult human heart [28]. The presence of fibrotic tissues in the heart engenders the loss of pump function and circulatory deficiency, thereby promoting heart failure. Therefore, inhibiting excessive cardiac fibrosis and shrinking the area of fibrosis post-MI are effective strategies for mitigating heart dysfunction and preventing heart failure.

In our study, we found that TGF-β, TGFBR1, α-SMA, and Col-1 were significantly elevated in the infarcted myocardium, which is consistent with previous studies [9].The TGF-β/Smad signaling specific to CFs allegedly underlies cardiac fibrosis [8]. TGF-β binds to TGFBR1 and TGFBR1 and then propagates intracellular signals downstream through Smad proteins, which are involved in the regulation of target genes expression. Besides, TGF-β stimulation induces the conversion of fibroblasts into myofibroblasts and enhances the synthesis of extracellular matrix proteins, promoting ventricular remodeling post-MI [9].

Many studies have found that miRNA is crucial in cardiovascular diseases [11–15]. It has also been shown that miR-130a targets on PDE4D to regulate apoptosis in cardiomyocytes [23]. However, no studies have shown its specific role in cardiac fibrosis post-MI. In our study, we found that miR-130a expression was significantly reduced in infarcted and fibrotic myocardium. We conjecture a potential link between miR-130a and myocardial infarction. We, therefore, wanted to determine the role of miR-130a in cardiac fibrosis post-MI and to explore the specific mechanism. To do so, we constructed AAV-9 vector miR-130a to study the effect and mechanism of miR-130a on cardiac fibrosis post-MI in a mouse model of myocardial infarction. We found that overexpression of miR-130a statistically improved cardiac function and reduced cardiac fibrosis area in mice post-MI. It indicates that miR-130a has an inhibitory effect on cardiac fibrosis post-MI.

To identify the specific molecular mechanism of miR-130a, we conducted the eukaryotic transcriptome sequencing analysis, and the venn diagram isolated 6 genes down-regulated by miR-130a. Among the 6 genes enrolled, we observed a complementary binding site between miR-130a and TGFBR1 mRNA 3ʹUTR. TGFBR1, a receptor of TGF-β, is essential to fibrosis in the cardiovascular system and other organs [29]. Previous studies have shown that the cardiac fibroblast-specific deletion of TGFBR1 improves cardiac overload-instigated ventricular remodeling and dysfunction [8]. Therefore, we speculate that TGFBR1 might be a miR-130a target-acting molecule that regulates TGF-β/Smad signaling. A single microRNA can regulate many targeted genes, we carried out the dual-luciferase reporter assay, confirming that *Tgfbr1* was indeed a target gene of miR-130a. In the cell transfection experiments, miR-130a negatively regulates the TGF-β/Smad signaling. Taken together, we determined that miR-130a inhibits the activation of TGF-β/Smad signaling by targeting TGFBR1.

We also found the inhibitory effect of miR-130a on α-SMA and Col-1. α-SMA is the most commonly used molecular marker of myofibroblasts [30], which exhibit improved collagen protein secretion [31]. The phenotypic transformation of CFs into myofibroblasts is a key event in myocardial injury and cardiac remodeling processes [32]. Collagen proteins in the myocardium include types I, III, IV, V, and VI, with structural type I collagen the most abundant (>70%) [5]. To establish whether TGFBR1 is a key functional target of miR-130a in CFs, we co-transfected CFs with miR-130a and TGFBR1. Our findings revealed that co-transfection with TGFBR1 offset miR-130a’s inhibitory potency on α-SMA and Col-1 in CFs, consistent with one study’s revelation that the application of a TGFBR1 inhibitor reversed CFs differentiation [33]; this report, combined with our results, suggests that miR-130a obstructs CF conversion to myofibroblasts and collagen deposition through miR-130a’s targeting of TGFBR1.In conclusion, we conclude that miR-130a plays an important role in the post-infarction fibrosis process.

## 5 Conclusions

In summary, we have demonstrated that miR-130a exerts its antifibrotic properties by directly targeting TGFBR1 to regulate the activity of TGF-β/Smad signaling and inhibit the transformation of CFs to myofibroblasts. miR-130a is a promising therapeutic target for alleviating cardiac fibrosis post-MI and improving cardiac function. However, more in-depth studies must be run for clear and precise inferences.

## Supplementary Material

Supplemental Material
