# Supplementary figures and images for: MicroRNA-130a attenuates cardiac fibrosis after myocardial infarction through TGF-β/Smad signaling by directly targeting TGF-β receptor 1
Source: Bioengineered. 2022 Feb 21;13(3):5779–91. doi: 10.1080/21655979.2022.2033380 (PMC8973730; doi:10.1080/21655979.2022.2033380)

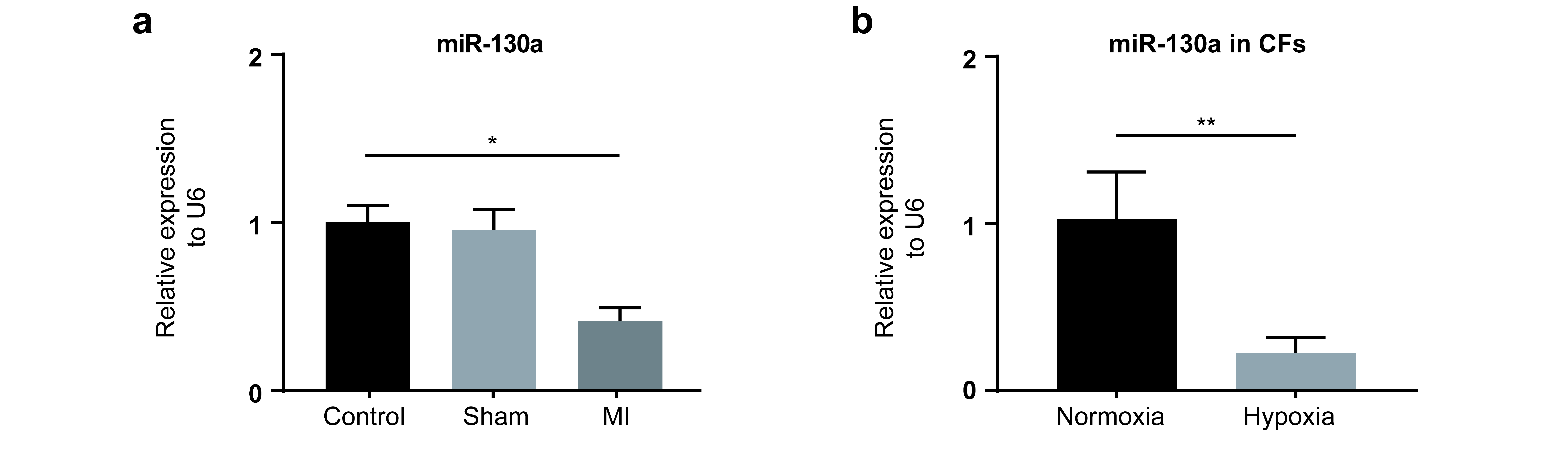

Supplement: Supplemental Material [file KBIE_A_2033380_SM5058.zip › supplementary/supplementary 1.tif]

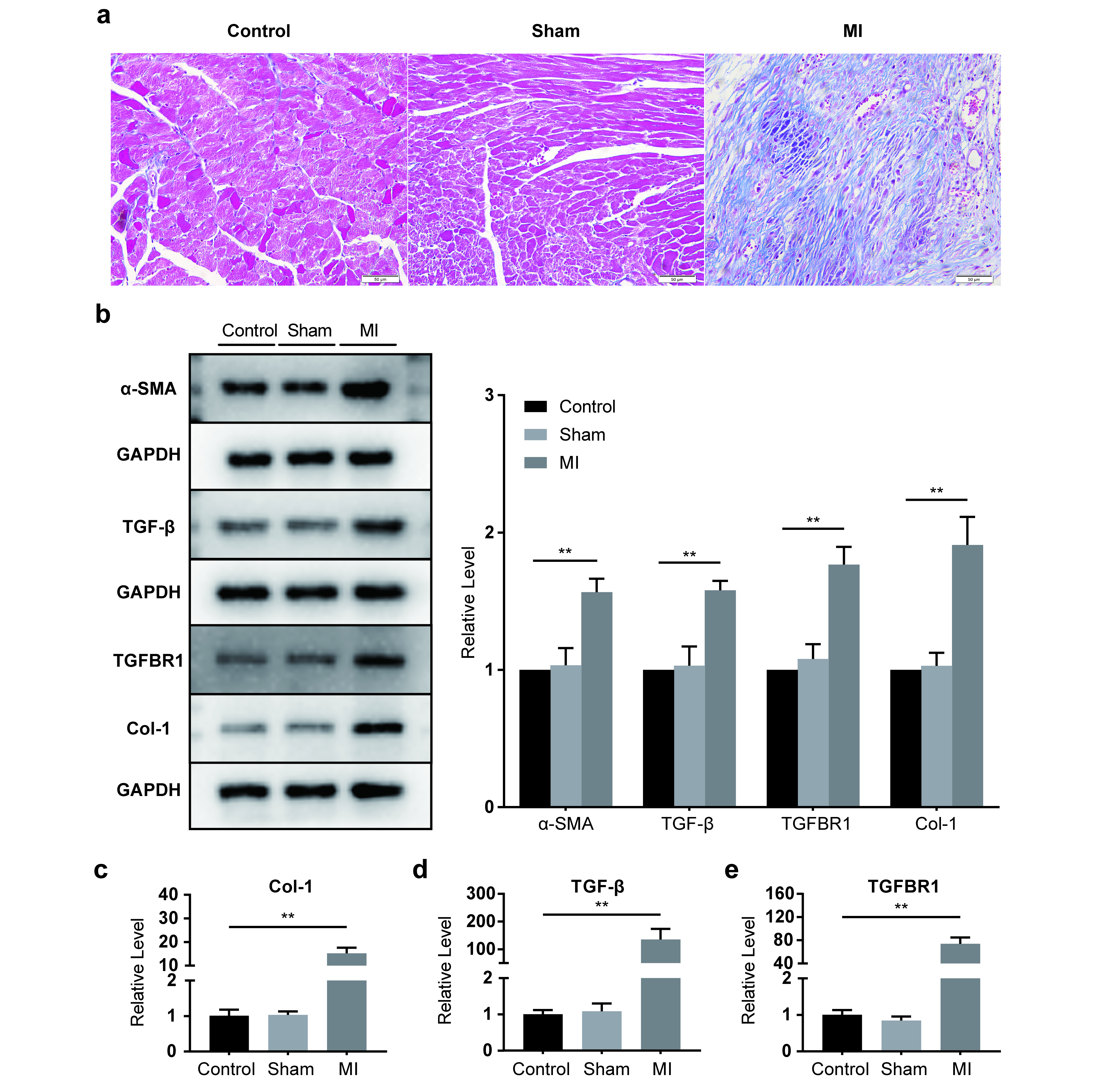

Supplement: Supplemental Material [file KBIE_A_2033380_SM5058.zip › supplementary/supplementary 2.tif]

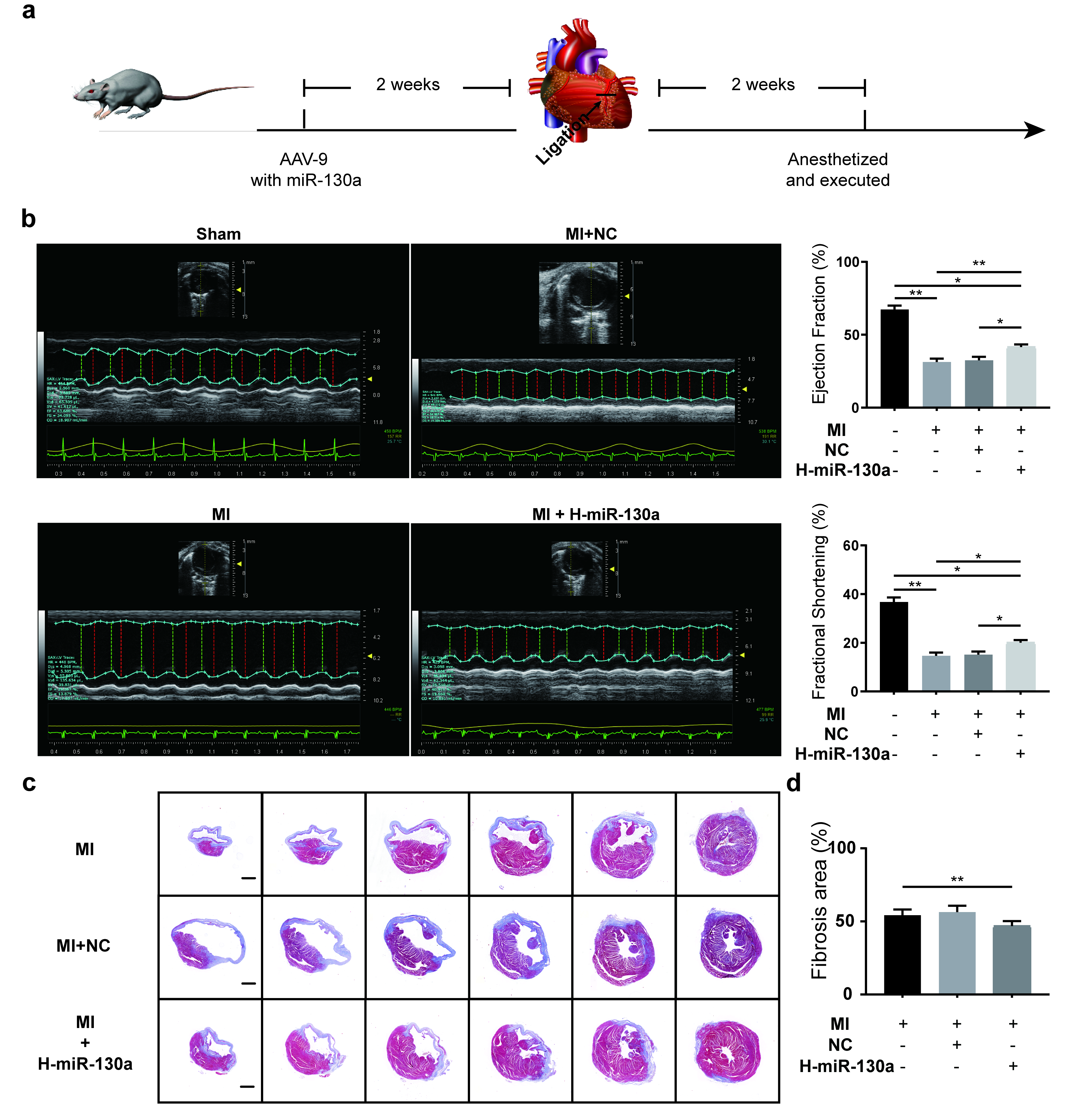

Supplement: Supplemental Material [file KBIE_A_2033380_SM5058.zip › supplementary/supplementary 3.tif]
